# Sex and age differences in self-reported immune fitness

**DOI:** 10.1016/j.bbih.2024.100792

**Published:** 2024-05-03

**Authors:** Kiki EW. Mulder, Pauline A. Hendriksen, Guusje A. Ulijn, Emina Išerić, Johan Garssen, Joris C. Verster

**Affiliations:** a Division of Pharmacology, Utrecht Institute for Pharmaceutical Sciences, Utrecht University, 3584CG, Utrecht, the Netherlands; b Global Centre of Excellence Immunology, Nutricia Danone Research, 3584CT, Utrecht, the Netherlands; c Centre for Mental Health and Brain Sciences, Swinburne University, Melbourne, VIC, 3122, Australia

**Keywords:** Sex, Age, Immune fitness, Immune status, Health perception

## Abstract

Studies have reported sex and age differences in self-rated health. On average, women rate their health as being poorer compared to men, and older individuals report poorer health than younger individuals. The current study evaluated sex and age differences for self-reported immune fitness, i.e. the capacity of the body to respond to health challenges (such as infections) by activating an appropriate immune response in order to promote health and prevent and resolve disease. Data from different survey studies (N = 8586) were combined for the current analyses. N = 8064 participants (93.3%) completed the single-item scale to assess momentary immune fitness (mean (Standard deviation, SD) age of 32.4 (16.7) years old, range: 18 to 103, 68.0% women) and N = 4263 participants (49.7%) completed the Immune Status Questionnaire (ISQ) to assess past year's immune fitness (mean (SD) age of 40.9 (17.1) years old, range: 18 to 103, 61.1% women). The analyses revealed that women rated their momentary and past year's immune fitness significantly lower than men (*p* < 0.001). A small but significant decline in momentary immune fitness when aging was found (r = −0.073, *p* < 0.001). In contrast, past year's immune fitness steadily improved with progressing age (r = 0.295, *p* < 0.001), and for each age group the difference from the 18–24 years old group was statistically significant (*p* < 0.001). When using age as covariate, the sex differences in immune fitness remained significant for both momentary immune fitness (*p* < 0.001) and past year's immune fitness (*p* < 0.001). In conclusion, women report a poorer momentary and past year's immune fitness than men. The sex effects in immune fitness are robust and seen across all age groups except the elderly. A relative stable momentary immune fitness was found across the age groups. However, past year's immune fitness (assessments with the ISQ) improved with age. This observation may be related to the fact that the studies comprised convenience samples. Therefore, the observed age effects should be interpreted with caution and require further investigation in nationally representative samples.

## 1 Introduction

Immune fitness can be defined as the capacity of the body to respond to health challenges (such as infections) by activating an appropriate immune response in order to promote health and prevent and resolve disease, which is essential for improving quality of life (Verster et al., 2023a). Over the past years, several scales and questionnaires have been developed to assess self-reported immune fitness, including a single-item assessment scale ranging from 0 (poor) to 10 (excellent), and the Immune Status Questionnaire (ISQ), a 7-item scale assessing the frequency of experiencing immune-related complaints (Verster et al., 2023a; Wilod Versprille et al., 2019). Whereas the ISQ is suitable only for retrospective assessments (e.g., covering past year), the single-item assessment can be used for both momentary and retrospective assessments. A growing body of international scientific literature has reported on the use of these assessment tools and linked immune fitness ratings to a variety of psychosocial- and health correlates. For example, reduced immune fitness has been associated with poorer sleep (Balikji et al., 2018a), an unhealthy daily diet (Van Oostrom et al., 2022), poorer mood (Balikji et al., 2022a), increased number and severity of the 2019 coronavirus disease (COVID-19) symptoms in individuals infected with severe acute respiratory syndrome coronavirus 2 (SARS-CoV-2) (Kiani et al., 2022a), poorer wound healing (Balikji et al., 2022b), abnormal body mass index (both under- and overweight) (Kiani et al., 2022b), reduced physical activity (Alharbi et al., 2023), increased alcohol consumption (Merlo et al., 2021), and reduced quality of life (Verster et al., 2021).

In addition to health effects, reduced immune fitness has also serious economic consequences in terms of increased absenteeism and presenteeism. For the Netherlands, it was estimated that the productivity of workers with reduced immune fitness was decreased by 22.8 % (Sips et al., 2023). The costs for the Dutch economy (in 2019) associated with absenteeism and presenteeism were estimated at 10.7 billion (Sips et al., 2023). It is thus understandable that there is an increasing interest in the assessment of immune fitness.

Whereas traditional biomarker assessments can be used to identify systemic inflammation, these assessments are often invasive (i.e., when using a blood draw), time-consuming, and relatively expensive techniques. In contrast, results of a self-reported assessment of immune fitness are readily available at minimal costs. As such, they can serve as a diagnostic screening tool, to identify individuals that may require further investigation. In addition to these practical advantages, reduced immune fitness is an important sign for individuals that action needs to be taken (e.g., a doctor's visit or lifestyle adjustment) to recover from this suboptimal health state.

The single-item immune fitness rating is a global assessment on a scale ranging from 0 (very poor) to 10 (excellent). Being a global assessment, this implies that an individual will take into account all factors that contribute to overall immune fitness. These include, but are not limited to, the frequency of occurrence, duration, and severity of various immune-related complaints, their impact on daily activities and quality of life, and current health status (Verster et al., 2023a, 2023b). In contrast, the ISQ assesses only the frequency of past year's occurrence of seven common immune-related complaints. The ISQ provides no information on other important factors related to overall immune fitness such as the severity of these immune-related complaints. Currently, there are no biomarkers available to assess overall immune fitness. However, there are several biomarkers available to assess systemic inflammation. Although the concept of systemic inflammation may be related to immune fitness, these biomarkers usually only alter in case of disease and significant reduced immune fitness. Given the latter, it is not surprising that a direct comparison of biomarkers of systemic inflammation (e.g., C-reactive protein, interleukins, and immunoglobulin A) and overall immune fitness ratings among relatively healthy young adults provided only modest correlations between the two (Mulder et al., 2023). In this context, it is important to note that individuals can report reduced perceived immune fitness while no changes are seen on biomarker assessments, as their immune fitness ratings remain within the ‘normal/healthy’ range. For example, a healthy athlete may have a usual immune fitness rating of 9, but experiences reduced immune fitness and then rates his immune fitness 7 out of 10). Alternatively, an individual with underlying disease is likely to have biomarker scores that are outside the normal range. These observations underline the importance of global assessments of perceived immune fitness.

Since self-reported immune fitness reflects an individual health perception, it may be influenced by patient characteristics, social desirability, and expectations, which are well-known confounders in sociological and psychological measurement theory (Verster et al., 2023a; Eriksson et al., 2001; Chiang et al., 2015). It is therefore likely that patient characteristics such as sex and age have an impact on the reporting of immune fitness. It is important to investigate these, as potential sex and age differences in perceived immune fitness may have implications for medical help seeking by patients and treatment compliance, and may aid the interpretation by health care workers of patient-reported immune fitness ratings.

With regard to sex, studies consistently found that self-rated health is poorer among women than men (Boerma et al., 2016a). Therefore, it is hypothesized that immune fitness will be rated significantly lower by women compared to men. Regarding age, national representative studies consistently revealed a gradual health decline when aging (Case and Deaton, 2003). This is understandable, as elderly individuals are more likely to have immune-related diseases than younger individuals (Jagger et al., 2008). As people age, physical fitness decreases making it harder to perform everyday activities, and may result in losing independence (Permanyer et al., 2022; Walston, 2012). Therefore, it is hypothesized that self-reported immune fitness will be poorer in older individuals. To further investigate these hypotheses, the current study aimed to evaluate possible sex and age differences in self-reported immune fitness.

## 2 Methods

Data from twelve studies conducted by our research group at Utrecht University that examined sex (male/female), age, and immune fitness were combined into one dataset (Kiani et al., 2022a; Verster et al., 2021; Otten et al., 2016; Mackus et al., 2017; Fernstrand et al., 2017; Becht et al., 2022; Balikji et al., 2018b; Huls et al., 2018; Baars et al., 2019a, 2019b; Sulzer et al., 2019; Van de Loo et al., 2020). The original studies obtained ethics approval (Study reference 25–31), or no formal ethics approval was required to conduct the survey according to the Dutch Central Committee of Research Involving Human Subjects (study reference 22–24). In all studies, all participants provided informed consent and approved the use of their data for scientific purposes. The studies had limited inclusion criteria, and no additional exclusion criteria. For all studies, except two student surveys (reference 25 and 31), the required minimum age to participate in the study was 18 years old. In a number of studies the only other inclusion criteria was being a student (reference 22–26), being a student that consumes alcohol (reference 31), being a young adult within the age range of 18–30 or 18–35 years old (reference 11 and 30, respectively), being a raw milk consumer (reference 28 and 29), having self-reported sleep complaints (reference 27), or being tested for SARS-CoV-2 (reference 6, ‘Corona test street’ (COTEST) study). For one study (reference 6, ‘Corona lockdown: how fit are you’ (CLOFIT) study), there were no specific inclusion or exclusion criteria, except being 18 years of age or older. For the CLOFIT study, baseline (2019, pre-pandemic data) was included in the pooled dataset. A summary of the individual studies and the demographics of participants is given in Table 1.

**Table 1:** Background information on the individual studies and their participants.

Ref.	Country	Year of data collection	Recruitment method	Survey format	Population	N	male/female ratio (%)	Age range (years)	Single item IF	ISQ
6	The Netherlands	2020	Facebook advertisement	Online	General population (CLOFIT study)	1910	36.0/64.0	18–94	Yes	Yes
6	The Netherlands	2020–2021	E-mail	Online	People tested for SARS-CoV-2 (COTEST study)	1084	54.5/45.5	18–88	Yes	Yes
11	Fiji	2018	Face-to-face	Paper	International young adults	333	43.5/56.5	18–35	Yes	Yes
22	The Netherlands	2016	Face-to-face	Paper	Students	779	37.6/62.4	18–30	Yes	No
23	The Netherlands	2015	Face-to-face	Paper	Students	410	44.4/55.6	18–30	Yes	No
24	The Netherlands	2014	Face-to-face	Paper	Students	509	28.1/71.9	18–30	Yes	No
25	The Netherlands	2019	Face-to-face	Paper	Students	291	44.0/56.0	17–32	Yes	Yes
26	The Netherlands	2016	Facebook advertisement	Online	Students	2498	16.6/83.4	18–26	Yes	No
27	The Netherlands	2018	Facebook advertisement	Online	Self-reported sleep complaints	1230	24.6/75.4	18–103	Yes	Yes
28	USA	2018	Internet announcement by farmers	Online	Raw milk consumers	380	33.0/67.0	19–86	Yes	No
29	The Netherlands	2018	Invitation leaflet on kefir bottle	Online	Raw milk kefir consumers	451	36.6/63.4	19–103	Yes	Yes
30	The Netherlands	2017	Social media	Online	Young adults	279	35.5/64.5	18–30	Yes	Yes
31	The Netherlands	2019	Face-to-face	Paper	Students who consume alcohol	198	26.3/73.7	17–25	Yes	No

Abbreviations: IF = immune fitness (momentary), ISQ = Immune Status questionnaire (past year), CLOFIT study = ‘Corona lockdown: how fit are you’ study, COTEST study = ‘Corona test street’ study.

No ethics approval was needed for the current pooled data analysis. All surveys collected data on the participants' biological sex (male or female) and age. Momentary immune fitness (at the moment of survey completion) was assessed using a single-item scale ranging from 0 (poor) to 10 (excellent) (Verster et al., 2023a, 2023c). Past year's immune fitness was assessed with the Immune Status Questionnaire (ISQ) (Wilod Versprille et al., 2019). The ISQ comprises the items ‘common cold’, ‘diarrhea’, ‘sudden high fever’, ‘headache’, ‘muscle and joint pain’, ‘skin problems (e.g. acne & eczema)’ and ‘coughing’. Participants answered how frequently they experienced these seven immune-related complaints. Answering possibilities were ‘never’, ‘sometimes’, ‘regularly’, ‘often’, and ‘(almost) always’. The overall ISQ score, after recoding (Wilod Versprille et al., 2019), ranges from 0 (poor) to 10 (excellent). The test-retest reliability of the single-item immune fitness rating and the ISQ are considered good, with test-retest correlations of r = 0.85 and r = 0.80, respectively (Wilod Versprille et al., 2019; Verster et al., 2023c).

The data were analyzed with SPSS (IBM Corp. Released, 2013. IBM SPSS Statistics for Windows, Version 29.0. Armonk, NY: IBM Corp.). A total of N = 10,352 individuals participated in the 12 studies. The purpose of the current pooled analysis was to evaluate possible sex and age differences in self-reported immune fitness. Participants were included in the pooled dataset if they were 18 years or older and reported age, sex, and completed the single-item immune fitness rating and/or the ISQ. The final dataset comprised N = 8586 participants. Mean and standard deviation (SD) were computed for each variable. Sex differences were tested for statistical significance with the Independent Samples Mann-Whitney *U* Test. Age differences were tested for significance with the Independent-Samples Kruskal-Wallis Test. Bonferroni's correction was applied to correct for multiple comparisons.

## 3 Results

N = 8064 participants completed the single item perceived immune fitness scale. The mean (SD) age of participants was 32.4 (16.7) years old (range: 18 to 103), and 68.0% of the sample were women. A subsample of N = 4263 participants completed the ISQ. The mean (SD) age of the subsample that completed the ISQ was 40.9 (17.1) years old (range: 18 to 103), and 61.1% of the subsample were women.

### 3.1 Sex

A summary of the data according to sex is presented in Table 2. Overall, women rated their momentary immune fitness significantly lower than men (*p* < 0.001). ISQ scores of women were also significantly lower than ISQ scores of men (*p* < 0.001).

**Table 2:** Immune fitness according to sex.

Sex	Immune fitness (momentary)		ISQ (past year)	
	N	Mean (SD)	N	Mean (SD)
Men	2575	7.8 (1.5)	1658	7.7 (2.2)
Women	5489	7.2 (1.7)*	2605	6.4 (2.6)*

Mean and standard deviation (SD) are shown. Significant differences between men and women (*p* < 0.05, 2-sided) are indicated by *. Abbreviations: N = number of participants, ISQ = immune status questionnaire.

**Fig. 1:**
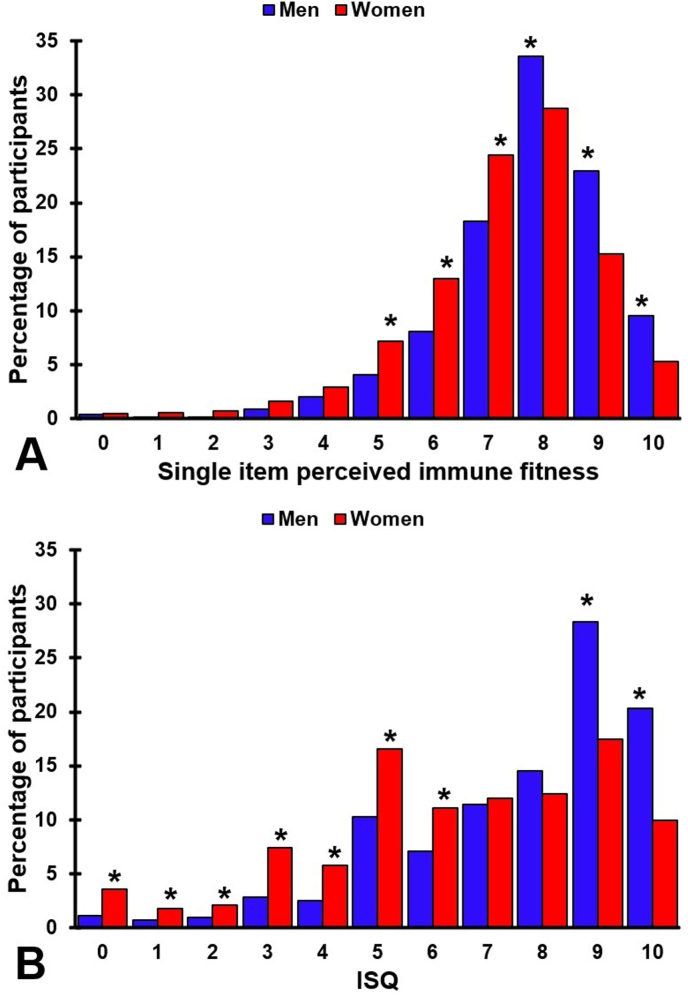
Immune fitness according to sex. Distribution of participants across the scale scores are shown for momentary (single item) immune fitness (Fig. 1a) and past year's immune status (ISQ) (Fig. 1b). Significant sex differences (*p* < 0.0045, after Bonferroni's correction for multiple comparisons) are indicated by *. Abbreviation: ISQ = immune status questionnaire.

The distribution of momentary (single-item) immune fitness scores and the ISQ scores for men and women are shown in Fig. 1. Significant higher percentages of women endorsed the single-item immune fitness scores of 5, 6, and 7 (*p* < 0.001), whereas significantly higher percentages of men endorsed immune fitness scores of 8, 9, and 10 (*p* < 0.001) (See Fig. 1a). In line, significant higher percentages of women had past year's immune fitness (ISQ) scores of 0–6 (*p* < 0.001), whereas significantly higher percentages of men had ISQ scores of 9 and 10 (*p* < 0.001) (See Fig. 1b).

### 3.2 Age

A small but significant decline in momentary immune fitness when aging was found (r = −0.073, *p* < 0.001). The data according to age is summarized in Table 3. Momentary immune fitness of the middle aged groups (35–64 years old) was significantly lower (*p* < 0.001) compared to the 18–24 years old group. Momentary immune fitness of the age groups of 65 years and older did not significantly differ from the 18–24 years old group. Past year's immune fitness (ISQ scores) steadily improved with progressing age (r = 0.295, *p* < 0.001), and for each age group the difference from the 18–24 years old group was statistically significant (*p* < 0.001).

**Table 3:** Immune fitness according to age.

Age range (years)	Immune fitness (momentary)		ISQ (past year)	
	N	Mean (SD)	N	Mean (SD)
18–24	4756	7.6 (1.4)	1165	6.0 (2.6)
25–34	974	7.5 (1.6)	750	6.6 (2.4) *
35–44	490	6.9 (2.0) *	479	6.7 (2.6) *
45–54	660	7.0 (2.0) *	680	7.2 (2.5) *
55–64	713	7.1 (2.0) *	715	7.7 (2.2) *
65–74	375	7.4 (1.9)	384	8.0 (2.1) *
≥75	95	7.1 (2.1)	89	8.3 (2.1) *
Overall	8063	7.4 (1.7)	4262	6.9 (2.5)

Mean and standard deviation (SD) are shown. Significant differences the age groups and the 18–24 year old group (*p* < 0.0083, 2-sided, after Bonferroni's correction for multiple comparisons) are indicated by *. Abbreviations: N = number of participants, ISQ = immune status questionnaire.

### 3.3 The interaction of immune fitness with sex and age

**Fig. 2:**
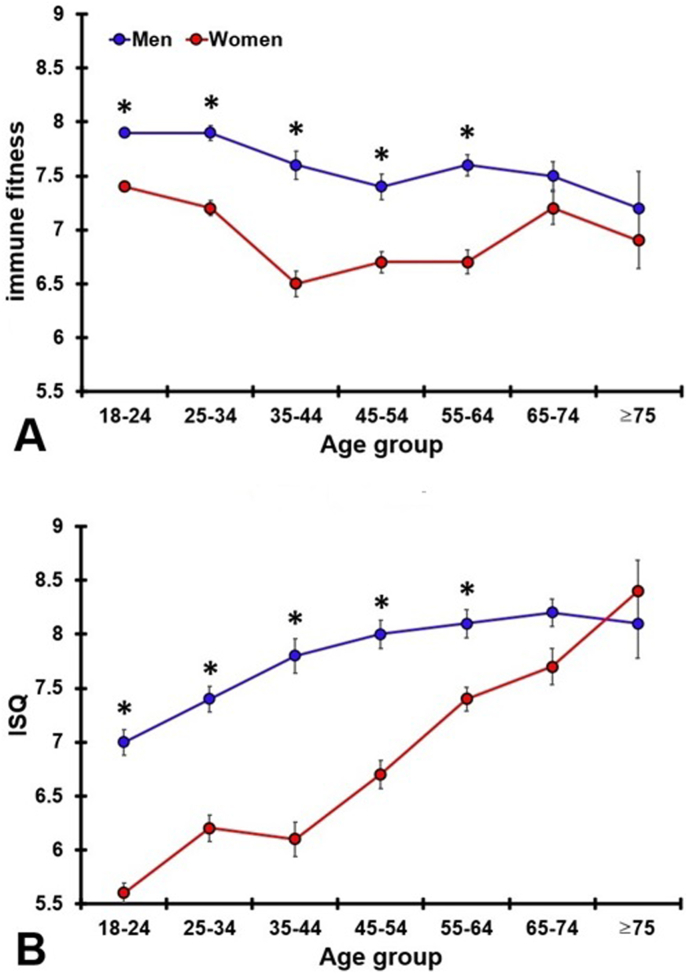
Immune fitness according to sex and age group. Data for men and women are shown for each age group for (a) mean (SD) momentary (single-item) immune fitness (Fig. 2a), and (b) the mean (SD) ISQ (Fig. 2b). Significant sex differences (*p* < 0.0045, after Bonferroni's correction for multiple comparisons) are indicated by *. Abbreviation: ISQ = immune status questionnaire.

Sex differences for each age group are summarized in Table 4 and Fig. 2. When using age as covariate, the sex differences in immune fitness remained significant for both momentary immune fitness (*p* < 0.001) and past year's immune fitness (*p* < 0.001). The differences were significant for all age groups, except for the elderly (65 years and older). Across the adult age groups (18–64 years old), the mean (SD) immune fitness scores of women were significantly lower than that of men.

**Table 4:** Sex differences according to age group.

Momentary (single-item) immune fitness					
Age range (years)	N		Mean (SD)		
	Men	Women	Men	Women	p-value
18–24	1249	3507	7.9 (1.3)	7.4 (1.4)	<0.001*
25–34	359	615	7.9 (1.4)	7.2 (1.7)	<0.001*
35–44	182	308	7.6 (1.7)^A^	6.5 (2.1)^A^	<0.001*
45–54	239	421	7.4 (1.9)^A^	6.7 (2.0)^A^	<0.001*
55–64	294	419	7.6 (1.7)^A^	6.7 (2.2)^A^	<0.001*
65–74	201	174	7.5 (1.8)^A^	7.2 (2.0)	0.353
≥75	51	44	7.2 (2.4)	6.9 (1.7)	0.085
Overall	2575	5489	7.8 (1.5)	7.2 (1.7)	<0.001*
**ISQ**					
	**N**		**Mean (SD)**		
**Age range (years)**	**Men**	**Women**	**Men**	**Women**	**p-value**
18–24	377	788	7.0 (2.3)	5.6 (2.5)	<0.001*
25–34	285	465	7.4 (2.1)	6.2 (2.5)^A^	<0.001*
35–44	183	296	7.8 (2.1)^A^	6.1 (2.7)^A^	<0.001*
45–54	254	426	8.0 (2.1)^A^	6.7 (2.6)^A^	<0.001*
55–64	304	411	8.1 (2.2)^A^	7.4 (2.2)^A^	<0.001*
65–74	209	175	8.2 (1.9)^A^	7.7 (2.3)^A^	0.031
≥75	46	43	8.1 (2.2)^A^	8.4 (1.9)^A^	0.785
Overall	1658	2605	7.7 (2.2)	6.4 (2.6)	<0.001*

Mean and standard deviation (SD) are shown. Significant differences the age groups and the 18–24 year old group (*p* < 0.0071, 2-sided, after Bonferroni's correction for multiple comparisons) are indicated by ^A^. Significant differences between men and women (*p* < 0.0083, 2-sided, after Bonferroni's correction for multiple comparisons) are indicated by *. Abbreviations: N = number of participants, ISQ = immune status questionnaire.

## 4 Discussion

Momentary and past year's immune fitness and the ISQ were evaluated in a large sample comprising N = 8586 participants between the age of 18 and 103. The analysis revealed significant sex differences for both momentary and past year's immune fitness. The differences were significant for all age groups, except the elderly (65 years and older). In the adult age groups, women consistently reported poorer immune fitness than men. This aligns with previous research on related self-reported health constructs such as general health (Boerma et al., 2016a). There could be two potential explanations for this observation. First, women experience chronic diseases, including autoimmune diseases, more often than men (Boerma et al., 2016a). Second, women might have a greater inclusiveness of symptoms in the perception of health than men, resulting in sex differences in the interpretation of being ill (Lekander et al., 2004). It has been shown that perceived health among men mainly reflects the presence of severe diseases and life-threatening health complaints, whereas health perception among women includes both (severe) diseases and non-life-threatening and minor health complaints (Lekander et al., 2004). In other words, when individuals are more inclusive in considering minor health complaints results, they tend to have lower immune fitness ratings than men. Third, social desirability and culturally rooted gender roles may contribute to these observed sex differences. For example, it has been suggested that men are often encouraged to present themselves as strong and independent, while women are encouraged to exhibit more nurturing and expressive behaviors (Benyamini et al., 2000; Eagly and Steffen, 1984). Men and women tend to self-characterize in ways consistent with these stereotypes (Eagly and Steffen, 1984). In line with this, women may be more inclined to report health issues and seek medical attention, while men may be more likely to downplay their symptoms and avoid seeking help (Eagly and Mladinic, 1989; Okamoto et al., 2008). Sex differences in self-presentation and communication styles can also influence how men and women answer health-related questions. Women may be more inclined to provide responses that align with social expectations, while men may be more likely to provide responses that accurately reflect their health status (Boerma et al., 2016b). Together, these factors may contribute to women reporting lower ratings of immune fitness compared to men.

Regarding age, a decline in momentary immune fitness was observed for the age groups 35–64 years old. However, although the effect was statistically significant, it was relatively small and not seen among the elderly groups. The findings are in line with a meta-analysis that found that global assessments of health, comparable to the single-item immune fitness rating, usually report poorer health for elderly and better health for younger respondents (Roberts, 1999). In contrast, an unexpected increase in past-year's immune fitness with aging, was observed. In contrast to the global single-item assessment of immune fitness, past year's immune fitness was assessed with the 7-item scale rating the frequency of occurrence of common immune related complaints. This observation is in disagreement with the fact that on average older individuals are more prone to developing immune-related diseases (Weyand and Goronzy, 2016; Haynes, 2020). It has been suggested that older adults are less reactive to unpleasant events, are better at ignoring irrelevant negative stimuli, and remember more positive knowledge than negative information. This could also be true for immune-related complaints. Alternatively, elderly may compare themselves to other elderly who are having a worse health status than themselves, and therefore report a relative better immune fitness than younger adults (Mather, 2012). Also, older individuals with a life-long experience of health and disease may be more conservative in labeling minor events as a health complaint. However, it is more likely that the way that participants were recruited and the study designs (mostly online surveys) resulted in convenience samples that do not comprise a nationally representative sample. Most of the studies recruited participants online via Facebook, on which elderly are underrepresented (Enzenbach et al., 2019). In addition, healthier elderly may be more likely to participate than unhealthier elderly (Golomb et al., 2012). These convenience sample characteristics may explain why past year's immune fitness improved with aging in the current sample. The observed age effects should therefore be interpreted with caution, and further research on age effects on immune fitness is needed using nationally representative samples instead of convenience samples.

A strength of the current study was its large sample size, which allowed well-powered analyses of sex and age effects. A first limitation of the study was that the pooled dataset consisted of convenience samples. Also, 11 of 13 samples originated from the Netherlands. Therefore, it is unclear to what extent our findings can be generalized to the general population. Future studies should aim at including nationally representative samples. Secondly, the purpose of the study was to evaluate possible sex and age differences in self-reported immune fitness. The purpose of the study was not to identify possible reasons *why* individuals may score high or low on immune fitness, such as underlying disease and health conditions. Information on underlying diseases or health conditions were not, or not consistently, recorded by the individual studies. However, such information could have been helpful for the interpretation of our findings. Future research should therefore also evaluate the possible causes of reporting immune fitness levels. Finally, the included studies did not assess biomarkers or other physiological effects. While there are no biomarkers for immune fitness, biomarkers of systemic inflammation could provide supportive evidence to determine the immune status of an individual. Previous research assessing biomarkers of systemic inflammation found both sex differences and age effects (Olivieri et al., 2023; Khera et al., 2005; Ershler and Keller, 2000; Wener et al., 2000). Also, the level of sex hormones has a relevant impact on immune functioning (Ter Horst et al., 2016). Including these assessments in future research will help interpreting sex and age effects in self-reported immune fitness.

The clinical implications of our findings are evident. The data demonstrate that health perception and reporting health complaints, including rating ones immune fitness, differ between the sexes and age groups. Healthcare workers should take these differences into account, and if necessary further inquire about the background reasons for patients’ health perceptions, and to whom they refer (e.g., using a same age group, same sex, a population with the same disease status, or the general population as reference group) when rating their health.

## 5 Conclusion

Women report a poorer momentary and past year's immune fitness than men. The sex effects in immune fitness are robust and seen across all age groups except the elderly. A relative stable (self-perceived) momentary immune fitness was found across the age groups. However, past year's immune fitness (assessments with the ISQ) improved with age. In particular due to the fact that the included studies comprised convenience samples, the observed age effects should be interpreted with caution.

## Funding

This research received no external funding.

## Institutional review board statement

Not applicable.

## Informed consent statement

Not applicable.

## CRediT authorship contribution statement

**Kiki EW. Mulder:** Writing – review & editing, Writing – original draft, Conceptualization. **Pauline A. Hendriksen:** Writing – review & editing, Conceptualization. **Guusje A. Ulijn:** Writing – review & editing, Conceptualization. **Emina Išerić:** Writing – review & editing, Conceptualization. **Johan Garssen:** Writing – review & editing, Conceptualization. **Joris C. Verster:** Writing – review & editing, Writing – original draft, Formal analysis, Data curation, Conceptualization.

## Declaration of competing interest

Over the past 3 years, J.C.V. has acted as a consultant/advisor for 10.13039/501100003769Eisai, KNMP, Med Solutions, Red Bull, Sen-Jam Pharmaceutical, and Toast! J.G. is part-time employee of Nutricia Research and received research grants from 10.13039/501100001720Nutricia research foundation, 10.13039/501100004522Top Institute Pharma, Top Institute Food and Nutrition, GSK, 10.13039/501100003958STW, NWO, Friesland Campina, 10.13039/100020214CCC, Raak-Pro, and 10.13039/100006939EU. The other authors have no potential conflicts of interest to disclose.

## Data Availability

Data will be made available on request.
